# Physicochemical Properties of Granular and Gelatinized Lotus Rhizome Starch with Varied Proximate Compositions and Structural Characteristics

**DOI:** 10.3390/foods12234330

**Published:** 2023-11-30

**Authors:** Xinyu Jiang, Yiting Gu, Lichao Zhang, Jinjian Sun, Jianan Yan, Ce Wang, Bin Lai, Haitao Wu

**Affiliations:** 1 State Key Laboratory of Marine Food Processing and Safety Control, National Engineering Research Center of Seafood, Collaborative Innovation Center of Seafood Deep Processing, School of Food Science and Technology, Dalian Polytechnic University, Dalian 116034, China; jiang_403@163.com (X.J.); 15142246566@163.com (Y.G.); zlc@sxu.edu.cn (L.Z.); yjn3vv@163.com (J.Y.); wangceyx@163.com (C.W.); laibin@dlpu.edu.cn (B.L.); 2 Institutes of Biomedical Sciences, Shanxi University, Taiyuan 030006, China; 3 Dalian Center for Food and Drug Control and Certification, Dalian 116037, China; 117187707@163.com

**Keywords:** lotus rhizome starch, starch granule, gelatinization, physicochemical properties, structural characteristics, correlation analysis, different region

## Abstract

As a traditional and popular dietary supplement, lotus rhizome starch (LRS) has health benefits for its many nutritional components and is especially suitable for teenagers and seniors. In this paper, the approximate composition, apparent amylose content (AAC), and structural characteristics of five LRS samples from different regions were investigated, and their correlations with the physicochemical properties of granular and gelatinized LRS were revealed. LRS exhibited rod-shaped and ellipsoidal starch granules, with AAC ranging from 26.6% to 31.7%. LRS-3, from Fuzhou, Jiangxi Province, exhibited a deeper hydrogel color and contained more ash, with 302.6 mg/kg iron, and it could reach the pasting temperature of 62.6 °C. In comparison, LRS-5, from Baoshan, Yunnan Province, exhibited smoother granule surface, less fragmentation, and higher AAC, resulting in better swelling power and freeze-thaw stability. The resistant starch contents of LRS-3 and LRS-5 were the lowest (15.3%) and highest (69.7%), respectively. The enzymatic digestion performance of LRS was positively correlated with ash content and short- and long-term ordered structures but negatively correlated with AAC. Furthermore, the color and network firmness of gelatinized LRS was negatively correlated with its ash content, and the retrograde trend and freeze-thaw stability were more closely correlated with AAC and structural characteristics. These results revealed the physicochemical properties of LRS from different regions and suggested their advantages in appropriate applications as a hydrogel matrix.

## 1. Introduction

Starch is widely used in food manufacturing as a thickener, stabilizer, or gelling agent to provide consistency and generate attractive texture. Differences in starch composition and structure lead to different properties that often vary with region and variety; these properties contribute to the various applications of starch. In addition, changes in structure and composition are generally related to the digestibility and glycemic index of starch [1]. By comparing the properties of seven purple sweet potato varieties, Yong et al. found that the starch variety greatly influenced the granule size, amylose content, short-range order, relative crystallinity, *in vitro* digestibility, and gelatinization properties of the starch [2]. Many factors, such as differences in the crop growth environment and processing methods, affect the composition of starch, thus resulting in different physicochemical characteristics [3,4,5]. Even the amylose content, crystallinity, and swelling ability of starches from the same botanical variety may vary according to their geographical origins and cultivation conditions [6,7,8,9]. Typically, amylose content in starchy foods is positively correlated with resistant starch content and negatively correlated with glycemic index [10]. Therefore, the detection of these indicators is important to understand the health benefits of starch materials. Moreover, as a crucial characteristic of starch, the properties of gelatinization and paste are directly related to their functionality, which influences the production reliability, stability of products, and consumer acceptance [11]. In general, the gelatinization properties of starches are the result of a combination of factors, including plant origin, granule morphology, amylose content, and relative crystallinity, which can lead to different industrial applications [12].

Lotus (*Nelumbo nucifera* Gaertn.) is widely cultivated in Asia. After thousands of years of artificial cultivation in China, more than 200 cultivars of lotus with edible rhizomes are available [13]. Normally, lotus rhizome is a kind of edible aquatic vegetable that contains fresh tissue and is rich in nutrients (including starch, protein, dietary fiber, flavonoid, polyphenol, and mineral nutrients), providing considerable health benefits [14]. The nutritional components in lotus rhizomes produced in different regions probably vary due to objective factors, such as geography, climate, and cultivar. As a result, the properties of the lotus rhizome starch (LRS) from those regions will also be varied. LRS is a traditional dietary supplement created from mature lotus rhizomes that have been consumed as a dessert for hundreds of years and provide health benefits, especially for teenagers and seniors [15,16]. In recent years, people have increased their requirements for nutrition, health, delicacy, and diversity as the modern food industry has rapidly developed. Currently, LRS is widely popular as a high-quality starch product with a unique health-preserving effect [17,18]. In the literature on LRS, Yu et al. compared the powder structure and gelatinization properties of LRS from two lotus cultivars but did not analyze the correlation between structure and physicochemical properties [19]. The physicochemical properties of LRS and lotus seed starch have been compared, including granule morphology, amylose content, structural characteristics, pasting properties, and hydrolytic properties [20]. Moreover, LRS exhibits distinctive properties compared with starch from other biological sources. For instance, Zhu reviewed the structure, properties, and applications of LRS and concluded that LRS contains large granules, is not greatly hydrolyzed, and is gluten-free [21]. Zhong et al. compared the physicochemical properties of LRS and kudzu root starch and found that LRS exhibits stronger thermal and mechanical shear stability and higher clarity during the cooking process [16]. Based on these findings, LRS would be an adequate food source to meet the health needs of individuals who follow a gluten-free diet and who suffer from celiac disease or overweight. However, the primary research on LRS is not comprehensive and thorough. To the best of our knowledge, the microstructure and hydrogel properties of the LRS after gelatinization have not been revealed and compared. In previous studies, reports on the mineral content in LRS and its effect on starch quality-related physicochemical properties were lacking. Therefore, a hypothesis was developed and demonstrated in this work based on experimental observations, which would fill the gap in LRS research.

The main objectives of this work were to determine the proximate composition and granular structure characteristics that affect the physicochemical properties of LRS before and after gelatinization. This was explored to increase knowledge on LRS and its consumption or utilization in food preparation and production. Moreover, the physicochemical characteristics of five LRS samples from different regions were compared, and the correlations between composition, structural characteristics, and physicochemical properties were analyzed. Consequently, this study could provide a wealth of detailed information to help guide the selection and application of LRS in daily consumption and the food industry.

## 2. Materials and Methods

### 2.1. Materials and Reagents

The original sources of LRS samples in this study were consistent with their commercial source. LRS-1 was procured from Hangzhou Sanjiacun Lotus Rhizome Powder Factory in Zhejiang Province, China; LRS-2 was procured from Hangzhou Ouxiangzhai Food Co., Ltd. in Hangzhou, China; LRS-3 was procured from Guangchang Lianxiang Food Co., Ltd. in Fuzhou, China; LRS-4 was procured from Hubei Meiweijia Refined Food Co., Ltd. in Huanggang, China; and LRS-5 was procured from Yunnan Huangnitang Lotus Industry Technology Development Co., Ltd. in Baoshan, China. The chemicals used were of analytical grade.

### 2.2. Proximate Composition Analysis

Proximate compositions, such as moisture (ambient pressure drying), protein (Kjeldahl determination), fat (Soxhlet extraction), and ash (dry ashing) contents, were measured following a modified version of the standard procedure (AOAC, 2010) [22]. The starch contents of the LRS samples were detected by an Amylum Content Assay Kit (Sangon Biotech (Shanghai) Co., Ltd., Shanghai, China). The metal content in five samples was detected by inductively coupled plasma-optical emission spectrometry [23].

### 2.3. Apparent Amylose Content (AAC) Analysis

The AAC in LRS samples was measured by an improved method [24]. The sample (10 mg, dry basis, DB) was dispersed in 5 mL of urea dimethyl sulfoxide containing nine parts DMSO and one part 6 M urea. The mixture was bathed in 95 °C water for one hour, supplemented by intermittent vortices. A 1 mL aliquot of the uniform mixture and 1 mL of iodine reagent (2.0% KI and 0.2% I_2_, *w*/*v*) were volumetric to 50 mL with deionized water, mixed immediately, and stored in darkness for 20 min. The AAC was evaluated from the absorbance at 620 nm. The recorded values were converted to percent of amylose by referencing a standard curve prepared with purified amylose from potato (A0512, Sigma-Aldrich) and amylopectin from corn (10,120, Sigma-Aldrich, St. Louis, MO, USA).

### 2.4. Morphology Observation

The granule morphology of five LRS samples was characterized by a scanning electron microscope (JSM-7800F, JEOL Ltd., Akishima, Japan). The sample powders were fixed at a conductive adhesive tape, sputter-coated with gold, and then observed at 2 kV accelerating voltage. Images of LRS granules at ×500 and ×2000 magnification were observed and collected.

### 2.5. Color Evaluation

The colors of granular and gelatinized LRS samples were determined using an UltraScan PRO spectrophotometer (Hunter Lab, Reston, VA, USA) following CIE guidelines. The whiteness value was calculated from Equation (1) [25]:

(1) $$Whiteness=100-\sqrt{{\left(100-L*\right)}^{2}+{a*}^{2}+{b*}^{2}}$$

### 2.6. In Vitro Enzymatic Digestion Analysis

The enzymatic digestibility of the LRS samples *in vitro* was determined by a method proposed by Englyst et al. [26], with modifications following a procedure by Wang et al. [27]. LRP (200 mg, DB) was dispersed in 15 mL of sodium acetate buffer (0.5 M), and then 10 mL of freshly prepared enzyme solution containing α-amylase (S31302, Shanghai Yuanye, Shanghai, China), amyloglucosidase (A7095, Sigma-Aldrich), and pancreatin (P3292, Sigma-Aldrich) was added. The digestion mixture was incubated for 2 h at 100 rpm in an incubator shaker at 37 °C. One aliquot (0.2 mL) was taken every 20 min (0, 20, 40, 60, 80, 100, 120). Absolute ethanol (0.8 mL) was used to dilute the hydrolysate and inactivate the enzymes. The amount of glucose released was measured with the GOPOD kit (D799408, Sangon Biotech) and multiplied by 0.9 to convert the percentage of hydrolyzed starch. Depending on the rate of hydrolysis, LRS was categorized as rapidly digestible starch (RDS), slowly digestible starch (SDS), or resistant starch (RS).

### 2.7. Fourier Transform Infrared (FTIR) Measurement

FTIR measurements of LRS samples were performed with a FTIR spectrometer (PerkinElmer Co., Ltd., Waltham, MA, USA). Spectra were scanned at a resolution of 4 cm^−1^, with 32 scans collected in the spectral range of 4000 cm^−1^ to 400 cm^−1^. The FTIR spectra of LRS samples were deconvolution processed using OMNIC software v8.2, and the linear shape was assumed to be Lorentzian. Thus, the absorbance ratios at 1045/1022 cm^−1^ and 1022/995 cm^−1^ could be calculated.

### 2.8. XRD Measurement

The crystalline patterns of five LRS samples from 5° to 40° (2 θ) were obtained using an X-ray diffractometer (XRD-7000, Shimadzu Corporation, Kyoto, Japan) with a scanning speed of 2°/min and a step size of 0.02° as the X-ray source at 30 mA and 40 kV. The relative crystallinity (RC) of the LRS samples was calculated by MDI Jade software v6.

### 2.9. Water and Oil Retention Capacity Analysis

The suspensions of 10% (*w*/*v*, DB) LRS in water or oil were gently stirred at ambient temperature for 30 min in preweighed centrifuge tubes, followed by centrifugation at 3500× *g* for 10 min. The water and oil retention capacities were calculated using Equations (2) and (3), respectively:

(2) $$\mathrm{Water}\mathrm{retention}\mathrm{capacity}\left(g/g\right)=\frac{\mathrm{Weight}\mathrm{of}\mathrm{water}\mathrm{absorbed}}{\mathrm{Weight}\mathrm{of}\mathrm{sample}\;(DB)}$$

(3) $$\mathrm{Oil}\mathrm{retention}\mathrm{capacity}\left(g/g\right)=\frac{\mathrm{Weight}\mathrm{of}\mathrm{oil}\mathrm{absorbed}}{\mathrm{Weight}\mathrm{of}\mathrm{sample}\;(DB)}$$

### 2.10. Swelling Power and Solubility Analysis

The 2% (*w*/*v*, DB) LRS suspensions were heated at 50, 60, 70, 80, and 90 °C for 30 min in a water bath with regular stirring. The sample was then naturally cooled and centrifuged (6000× *g*, 15 min), and the sediment and supernatant were separated. The swelling power and solubility were calculated by Equations (4) and (5), respectively:

(4) $$\mathrm{Swelling}\mathrm{power}\;(g/g)=\frac{\mathrm{Weight}\mathrm{of}\mathrm{sediment}\mathrm{after}\mathrm{centrifugation}}{\mathrm{Weight}\mathrm{of}\mathrm{sample}\;(DB)}$$

(5) $$\mathrm{Solubility}\left(\%\right)=\frac{\mathrm{Weight}\mathrm{of}\mathrm{dried}\mathrm{supernatant}}{\mathrm{Weight}\mathrm{of}\mathrm{sample}\;(DB)}\times 100\%$$

### 2.11. Differential Scanning Calorimetry (DSC) Measurements

The gelatinization of LRS suspensions was measured via a differential scanning calorimeter (DSC250, TA Instruments, New Castle, DE, USA). The 3 mg LRS sample (DB) was sealed with 6 μL deionized water in the sample pan and equilibrated for 12 h. The sample pans and the empty reference pan were heated from 30 to 100 °C at a rate of 10 °C/min.

### 2.12. Pasting Properties Analysis

Following a procedure by Jin and Xu [28], the pasting properties of LRS samples were determined by a rheometer (Discovery HR-1, TA Instruments, New Castle, DE, USA) equipped with a 40 mm diameter parallel plate. The LRS slurry (10%, *w*/*v*, DB) went through a programmed heating-cooling cycle as follows: heat at 25 °C for 60 s, heat from 25 °C to 100 °C at 10 °C/min, hold at 100 °C for 600 s, cool to 25 °C at 10 °C/min, and store at 25 °C for 60 s. The shear rate and gap were set at 200 s^−1^ and 500 μm, respectively.

### 2.13. Gel Microstructure Observation

The LRS samples were dispersed in deionized water to create suspensions (8%, *w*/*v*, DB), heated at 95 °C, and stirred regularly. After cooling, LRS gels were prepared. The gel microstructure of LRS was photographed by a cryogenic transfer scanning electron microscopy system (cryo-SEM). After the LRS gel sample was pre-frozen in liquid nitrogen slush, it was transferred to the cooled stage of the preparation chamber (PP3010T cryo-SEM preparation system, Quorum Technologies Ltd., Lewes, UK) under vacuum. Frozen samples were subjected to fracture, sublimation (−70 °C, 20 min), and sputtering Pt coating followed by imaging in the cryo-stage SU8010 SEM (Hitachi Co., Ltd., Chiyoda City, Japan). Moreover, the cryo-SEM images were analyzed using AngioTool software v0.5 for quantitative network analysis [29].

### 2.14. Light Transmittance Testing

LRS was dispersed in water and heated at 95 °C for 30 min with regular agitation to prepare the suspension (1%, *w*/*v*, DB). The samples were stored at 4 °C for 5 days, and the turbidity was measured and recorded at 640 nm UV absorbance every 24 h [30].

### 2.15. Freeze–Thaw Stability Analysis

The freeze–thaw stability of starch materials is directly related to their application in frozen food [31]. In addition, repeated freeze–thaw cycles may contribute to lower digestibility and health benefits of starch hydrogels [32]. The suspension of LRS (6%, *w*/*v*, DB) was heated to 95 °C for 30 min with regular agitation. The gel samples obtained were thawed in a 30 °C water bath for 3 h after 24 h of freezing at −18 °C and centrifuged at 6000× *g* for 15 min to remove the supernatant. Then, the syneresis was calculated using Equation (6). The number of freeze–thaw cycles was set to 5 in this experiment.

(6) $$Syneresis\left(\%\right)=\frac{\mathrm{Weight}\mathrm{of}\mathrm{separated}\mathrm{water}}{\mathrm{Weight}\mathrm{of}\mathrm{LRP}\mathrm{gel}}\times 100\%$$

### 2.16. Statistical Analysis

Triplicate determinations were performed. The data were subjected to variance analysis and expressed as the means ± standard deviations. Multiple comparisons were performed between the two groups using the S-N-K method, and levels of *p* < 0.05 were considered significant. In addition, principal component analysis (PCA) and Pearson correlation were used to find the relationships between the studied variables, and all graphs were drawn with Origin software v9.8.

## 3. Results and Discussion

### 3.1. Proximate Composition and Apparent Amylose Content (AAC) of LRS Powder

Proximate analysis is usually an important part of assessment on the nutritional significance of starch products [33]. The proximate compositions of five LRS samples are shown in Table 1. The moisture content, starch content, and ash of the five LRS samples differed significantly (*p* < 0.05), and the ranges were 74.4–120.3 g/kg, 718.8–886.7 g/kg, and 3.5–11.8 g/kg, respectively. Among them, LRS-5 had the highest starch content, and LRS-3 contained a higher ash content. Compared to the other four, the starch content of LRS-5 and ash content of LRS-3 were 1.1–1.2-fold and 2.4–3.4-fold greater, respectively. This result indicated the possibility that LRS-5 exhibited higher starch purity, and LRS-3 exhibited lower starch purity. In addition to the significant starch components, LRS also contained tiny amounts of proteins, lipids, and beneficial metal elements (Table 2). The residual inorganic ash that is generated after starch is fully combusted usually represents the total mineral content [34]. Remarkably, calcium (354.5–486.3 mg/kg) was the most abundant metal element in the LRS. Oyeyinka et al. reported that bitter yam starches contain approximately 0.2 mg/100 g of calcium [35]. In contrast, the calcium content in LRS was considerable. In addition, the content ranges of iron and magnesium reached 50.6–302.6 mg/kg and 33.9–127.1 mg/kg, respectively; the highest values were attributed to LRS-3, which corresponded to the highest ash content in LRS-3. For manganese, the content in LRS was relatively low (only 2.2–7.2 mg/kg), and the highest value appeared in LRS-5. The proximate composition of LRS showed that it has good nutritional value and is a potential source of essential minerals, such as calcium and iron. Gluten-free foods have become more popular in recent years, but many gluten-free starches, including rice, corn, and potato starch, lack essential minerals, such as calcium, iron, magnesium, and manganese [36]. In contrast, LRS has certain nutritional advantages as a potential raw material for gluten-free foods. Obtaining iron from food is the most cost-effective strategy for the prevention of iron deficiency anemia and for benefiting women’s health [37]. One of the advantages of the LRS as a traditional dietary supplement is that it is rich in micronutrients for health benefits. Thus, LRS-3 might have potential applications in iron-fortified foods, depending on the ash content and major metal content of LRS samples. In addition, the color parameters of the LRS powder are presented in Table S1. LRS-3 was different from the other four samples because its L* and whiteness values were the lowest, which may be related to its higher iron and magnesium content. Similarly, Pietrzyk et al. found that iron and magnesium could cause a decrease in the brightness of starch [38].

The apparent amylose content (AAC) is the amylose and amylopectin long-chain (DP > 60) component content determined by the iodine-binding method, which is generally the key determinant of the processing, cooking, and eating quality of starch products [39]. In particular, AAC is a strong predictor of resistant starch, and higher levels of resistant starch generally mean that the fluctuations in blood sugar caused by the digestion of starchy foods are more gradual [40]. Therefore, it could be speculated that there would be specific health benefits associated with higher AAC in LRS, such as favoring the prevention of diabetes and cardiovascular disease [1]. In this work, the AAC in LRS ranged from 265.8 g/kg to 316.5 g/kg, as shown in Table 1. Among them, LRS-5 had the highest AAC, and LRS-2 had the lowest AAC. The results were consistent with previous reports that the amylose content of lotus rhizome starch was approximately 25–30% [41,42]. Shi et al. revealed a correlation between the structure and properties in millet starch, and the varied AAC values were highlighted [43]. Therefore, it is necessary to further explain the correlation between AAC and physicochemical characteristics in LRS.

### 3.2. Morphology of LRS Powder

**Figure 1:**
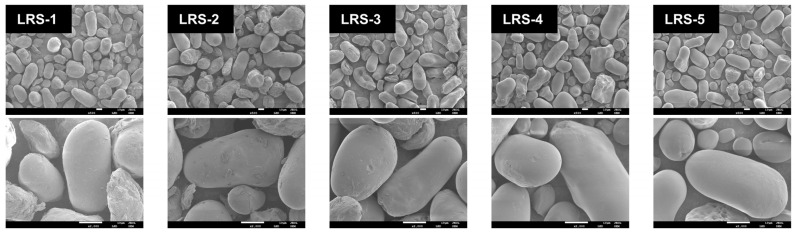
Scanning electron micrographs of five LRS samples. The magnifications were ×500 and ×2000, respectively.

The morphology of starch granules is among its basic physical properties and could be used to roughly distinguish the classification of starch [44]. The granule morphology of five LRS samples observed by SEM is presented in Figure 1. Our observation basically corresponded with previous research showing that LRS granules were mostly oval and elongated in shape, with measurements of 10–35 μm across and 10–50 μm long [45]. In the images with ×500 magnification, all the granules exhibited rod and oval shapes for large granules and near-spherical or irregular shapes for small granules. The surface heterogeneity of the LRS, with a hilum at one end of the elongated granules, is shown in micrographs [45,46]. Compared with the other four samples, the LRS-5 granule with higher AAC exhibited a smoother surface, showed a higher integrity, was less broken, and contained attached debris. This might occur because more amylose and long-chain starch tended to facilitate their interactions with amylopectin short-chain branching through entanglement, as found by Shi et al. [43]; thus, an enhanced frame structure occurs, which improved the compactness, rigidity, and volume strength of starch granules and maintained their integrity to a greater extent. Furthermore, the AAC as well as scraps, cracks, and fissures on the granule surfaces of the starch likely impact digestibility [43,47]. Therefore, it was necessary to further examine the digestive properties of LRS and to explore the structural causes.

### 3.3. In Vitro Enzymatic Digestive Properties of LRS Powder

**Figure 2:**
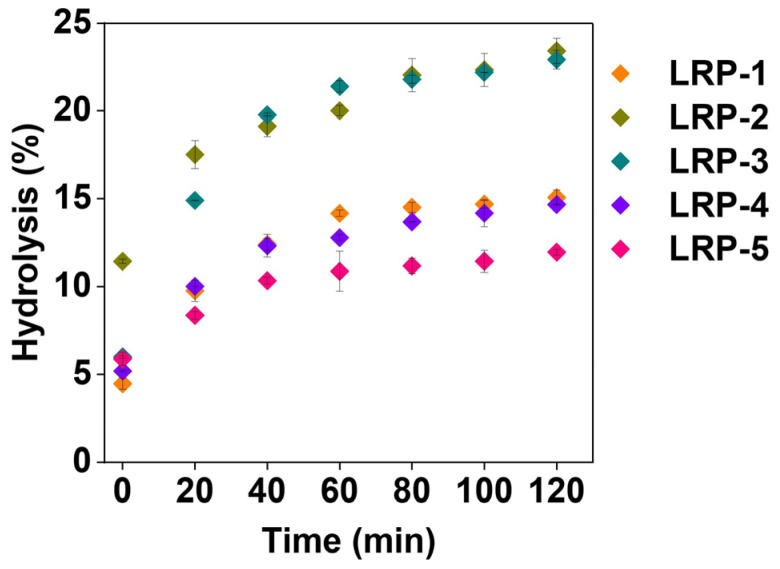
*In vitro* enzymatic digestion curves of five LRS samples.

The *in vitro* enzymatic digestive properties of the five LRS samples are presented in Figure 2. Digestion characteristics for all models showed an initial 40 min faster digestion phase followed by a slower digestion phase. In contrast, significant differences were observed in final digestion percentages at 2 h between samples, and the lowest was stained for LRS-5 (11.9%). As shown in Table 3, the RDS and SDS contents of the five LRS samples ranged from 12.4% to 40.6% and 17.9% to 40.1%, respectively. The RS content ranged from 15.3% to 69.7%, with the lowest in LRS-3 and the highest in LRS-5. These results indicated that LRS-5 was more resistant to digestion. The results obtained from the Pearson correlation analysis between the digestion characteristic components and ash or AAC of LRS are shown in Figure S1. In detail, the RS of LRS powder was negatively correlated with ash (−0.74749, *p* < 0.01) but positively correlated with AAC (0.66141, *p* < 0.01). The results indicated that LRS powder with high ash content had relatively poor anti-digestibility, and higher AAC content contributed to the resistance of starch to hydrolysis. Therefore, higher AAC in LRS might have specific health benefits in terms of delayed starch digestion. Moreover, it has been reported that high-amylose starch is less easily hydrolyzed than normal starch and waxy starch, which may result from the highly dense amorphous region in high-amylose starch granules due to the extensive interchain binding of amylose polymers [48,49]. Therefore, AAC and the amorphous region in LRS-5 might partly explain the slower rate and degree of LRS hydrolysis during enzymatic hydrolysis. Furthermore, it was necessary to characterize the short-range and long-range ordering of the five LRS samples.

### 3.4. Structural Characteristics of LRS Powder

**Figure 3:**
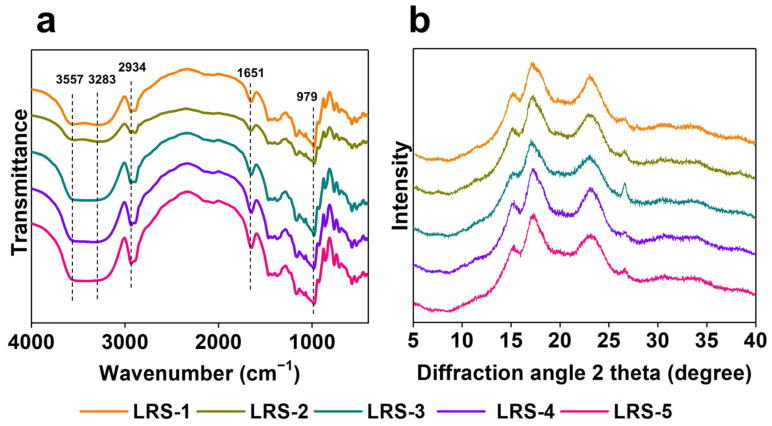
FTIR spectra (**a**) and XRD patterns (**b**) of five LRS samples.

The degree of short-range ordered structure in the outer region of starch was usually quantified by the ratio of absorbance (1045/1022 cm^−1^), and 1022/995 cm^−1^ was related to the ratio of amorphous to ordered structure [50]. According to the results of the FTIR spectra (Figure 3a), the C-H, C-O, and O-H vibration bands of the five LRS samples were not significantly different due to the similar chemical structure of the lotus rhizome starch. Furthermore, although the FTIR peak positions of the five LRS samples were almost the same, there were significant differences in the IR ratios of 1022/995 cm^−1^ and 1045/1022 cm^−1^, as shown in Table 4. LRS from different regions showed different short-range ordered and amorphous structures. In addition, the long-range ordered structure of LRS could be revealed by the relative crystallinity (RC) [51]. XRD was used to investigate the long-range order of the crystalline structures in five LRS powder samples. The diffraction patterns of the five LRS samples are presented in Figure 3b, and the calculated RC values are displayed in Table 2. Generally, the XRD pattern of starch exhibits spike and dispersion characteristics, representing crystalline and non-crystalline regions, respectively. The arrangement of starch granule crystals produces several polymorphs, and three of these polymorphs were expected (A-, B-, and C-type crystalline structures) [52]. As shown in Figure 3b, the five LRS samples presented a C-type crystalline structure characterized by firm peaks at 15°, 17°, and 23°. This finding was consistent with previous reports [20,53,54]. Moreover, the RC values of LRS samples ranged from 21.4% to 29.4%, with significant differences (Table 4). Among them, LRS-2, with the lowest AAC, showed the highest RC, and LRS-5, with the highest AAC, showed the lowest RC. As an important physicochemical parameter used to judge the cooking and dietary quality of starch products [55], ACC was significantly (*p* < 0.05) correlated with the structural properties of LRS (Figure S1). Specifically, AAC was significantly negatively correlated with R_1045/1022_ (Pearson correlation coefficient −0.93599) or RC (−0.95901) but positively correlated with R_1022/995_ (0.71926). As the crucial parameters of starch granules, the structural and crystal properties significantly influenced the physicochemical and functional properties of starch. It was suggested that LRS with higher AAC was characterized by lower short-range and long-range order as well as a higher amorphous region, which further contributed to the slower digestibility; this result has been confirmed with the millet starch data [43]. Overall, the digestion characteristics of LRS might result from a combination of factors, including ash content, scraps, cracks, and fissures on granule surface, ordered structure, etc. Lower ash content and smoother granule surface resulted in better resistance to digestion. Moreover, greater AAC and amorphous regions of LRS contributed to slower digestibility.

### 3.5. Water and Oil Retention Capacity of LRS Powder

**Figure 4:**
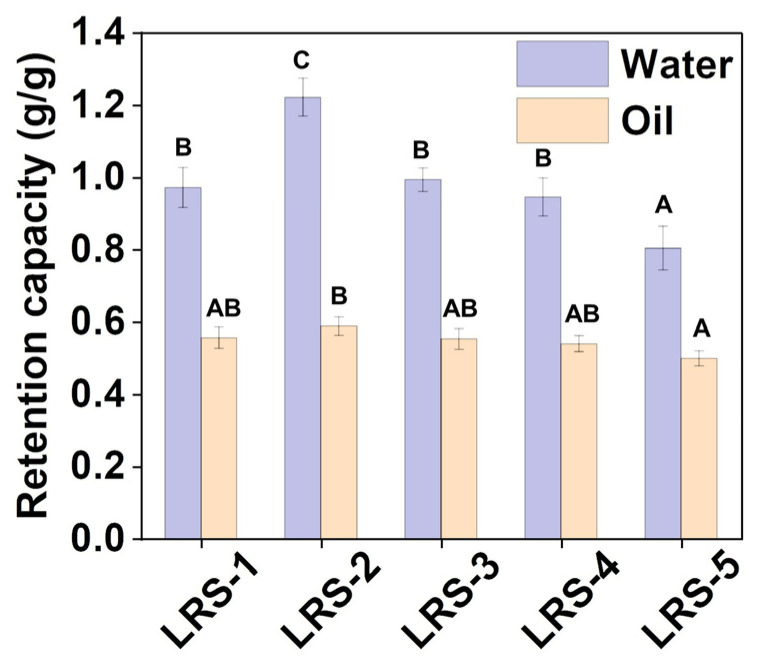
Water and oil retention capacity of five LRS samples. Different letters above the same color bars indicate significant differences (*p* < 0.05).

The water retention capacity, solubility pattern, and swelling behavior of starch granules are important for certain strategic applications in the food industry, such as fried bread, film, aerogel, etc., and are usually causal from structural properties [56,57,58,59]. As shown in Figure 4, the water retention capacity values of the LRS samples ranged from 0.80 to 1.22 (g/g), and the oil retention capacity values ranged from 0.50 to 0.59 (g/g). There was little difference in oil retention capacity among the five LRS samples. However, LRS-2 exhibited the highest water retention capacity, and LRS-5 showed the lowest, which was opposite to the trend observed for AAC. Similarly, Lopez Silva et al. have shown that the water retention capacity of corn starch when not gelatinized was negatively correlated with the content of amylose [60]. In addition, the water and oil retention capacities were positively correlated with the short- and long-range ordering of the LRS (Figure S1). Therefore, the water retention capacity of LRS granules might result from comprehensive factors. The higher short- and long-range order of LRS resulted in better water and oil retention capacity. A main reason could be that fewer hydroxyl groups were exposed by the surface molecules of starch granules that exhibited a less broken and smoother granule surface; as a result, the availability of water-binding sites decreased, and the water retention capacity changed [61]. Starches with higher water-holding capacity were used to improve the processing properties of some products, which helped to prevent the formation of lumps when mixed with other powders due to the slower water intake [62]. These findings could explain why the distinctive microstructural differences might affect the availability of water-binding sites in LRS powder samples [63].

### 3.6. Swelling Power and Solubility of LRS Powder

**Figure 5:**
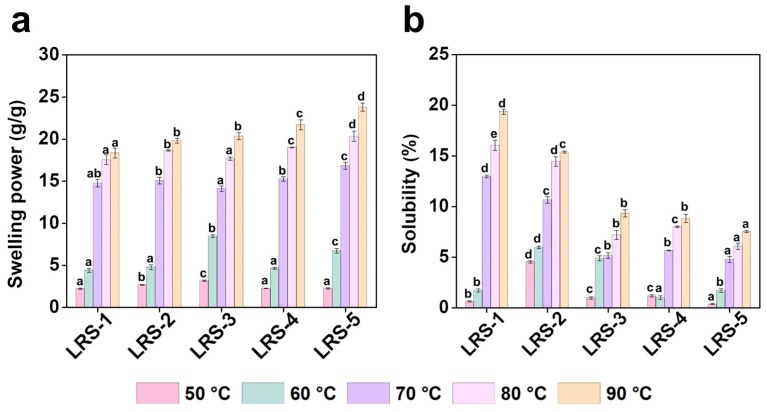
Swelling power (**a**) and solubility (**b**) of five LRS samples. Different letters above the same color bars indicate significant differences (*p* < 0.05).

Swelling power is an index used to measure the water-holding capacity of starch materials after they undergo gelatinization in the aqueous phase, and solubility reflects the water-soluble components during the gelatinization process [64]. The swelling power and solubility of LRS samples at various temperatures (50, 60, 70, 80, and 90 °C) are summarized in Figure 5a,b. At 50 °C, the difference in solubility among the five LRS samples was similar to their water retention capacity. Combined with the morphology of the LRS granules, this result could mainly result from the granule fragmentation (Figure 1). The swelling power and solubility were positively correlated with the temperature, and there was a sudden increase from 60 to 70 °C. Under normal conditions, the water solubility of starch at room temperature was very low. At 90 °C, the swelling power of the LRS samples varied from 18.4 g/g (LRS-1) to 23.8 g/g (LRS-5), and the solubility was 7.5% (LRS-5)-19.37% (LRS-1). These results suggested that LRS-5 exhibited the best swelling capacity and the minimum dissolution during swelling, reflecting the highest degree of interaction between starch chains in LRS-5 during gelatinization.

Remarkably, the short- and long-range order of LRS restricts the swelling of starch granules during gelatinization, which manifested as the negative correlation between R_1045/1022_ or RC and swelling power (Figure S1). A similar viewpoint was shown in a study on rice starch [55]. A possible reason for swelling power was that starch granules gradually absorbed water during heating and gelatinization, which destabilized the crystal structure and disordered the molecular orientation, leading to an increase in the interaction between starch chains in the amorphous and crystalline domains [65]. Therefore, the originally more ordered structure of starch granules contributed to the resistance to swelling. Ma et al. suggested that amylose and shorter branched-chain amylopectin chains included in AAC mainly helped form inner blocks of rice starch granules during gelatinization [66]. Therefore, the higher content of amylose and the lower order of microstructure might partially explain the higher swelling ability of LRS-5 [67]. In addition, the more intact granule structure increased the granule swelling and reduced the dissolution of components. Kumar et al. similarly found that the rough surface and agglomerates on starch were detrimental to the swelling index and gelatinization properties [68].

### 3.7. Thermal and Pasting Properties of LRS Powder

Thermal and pasting properties are important for starch application, especially in the food industry [69]. Moreover, the differences in AAC, starch granule morphology, and trace components explain the diversity of LRS in gelatinization behaviors, including thermal and pasting properties [70]. The thermal characteristic curves and parameters of five LRS samples obtained by DSC are presented in Figure S2a and Table 5, respectively. The gelatinization temperature of LRS ranged from 53 °C to 88 °C, which was consistent with that reported in other studies [18,48]. Within this range, starch granules undergo water absorption, swelling, rupture, and irreversible crystal structure destruction. The peak temperature (*T*_p_) of the five LRS samples ranged from 63.7 °C to 68.9 °C, which mainly resulted from double-helix crystallization dissociation [24]. Among the five samples, LRS-3 had the lowest values of onset temperature (*T*_o_), *T*_p_, and conclusion temperature (*T*_c_), indicating that a relatively low amount of energy is needed to initiate gelatinization; this could be related to the higher ash content of LRS-3 (Table 1) [71]. There was a significant negative correlation between *T*_o_ and ash content but no significant correlation between *T*_o_ and structural parameters, indicating that the onset temperature of the LRS gelatinization process might be mainly influenced by ash content rather than the ordered structure (Figure S1). A possible reason for this was that ash acted as a facilitator in the swelling of starch granules to produce viscosity. Nevertheless, the energy necessary for the gelatinization of the five LRS samples ranged from 4.8 to 6.1 J/g, which aligned with previous research [16]. The gelatinization enthalpy was positively correlated with AAC and R_1022/995_ but negatively correlated with R_1045/1022_ and RC, which reflected the variability of hydrogen bond alignment in LRS granules [65]. Therefore, it was mainly the AAC and amorphous structure of LRS that promoted the higher enthalpy. Furthermore, the gelatinization properties revealed by DSC affected by ash content could be reflected by pasting properties.

The pasting properties of starch are commonly quantified via the temperature-dependent viscosity of starch dispersion; these properties mainly depend on certain factors, such as RC, swelling power, amylose leaching, and friction between swollen granules [28]. The pasting properties of the LRS samples obtained from the rheometer are shown in Figure S2b,c. With increasing temperature, starch granules gradually gelatinized, and the viscosity within 60–80 °C increased sharply. At higher temperatures, the viscosity dropped slightly as the thermal motion accelerated. During the cooling process, the viscosity increased again due to the increased friction between swollen granules. The pasting temperature (PT), peak viscosity (PV), hot paste viscosity (HPV), cold paste viscosity (CPV), breakdown viscosity (BDV), and setback viscosity (SBV) of the five LRS samples are shown in Table 6. The PT and PV of the five samples evaluated range from 62.6 to 69.9 °C and 1.3 to 2.3 Pa·s, respectively. Compared with the others, gelatinizing LRS-4 was more challenging, as it involved the highest PT and the longest gelatinization time [72]. The pasting properties are important for evaluating the thickening and bonding functions of LRS used in the food industry. In terms of viscosity, LRS-5 exhibited the highest PV, with no apparent decrease in viscosity as heating continued, and it showed a higher final viscosity after cooling. In general, the PV value corresponds to the peak of starch granule expansion during gelatinization, and it is possible to correlate the matrix composition and molecular structure of starch [62]. In addition, the PV value can indicate the thick load in the mixing of ingredients, which exerts an important effect on the texture and functional properties of the products [73]. In this work, no significant correlation was observed between PV and AAC or short- and long-range ordered structures (Figure S1). Similar results were found for other starches [65,74,75]. The correlation between AAC and PV or PT for other starches was not consistent, and there could be many combinations and uncertainties [55]. Therefore, the PV and PT of starch might be more affected by impurities, such as ash. In addition, different studies using different samples of starch from the same species might exhibit different compositional/structural-characteristic correlation patterns, as the correlations between the starch characteristic parameters of the same species were particularly susceptible to relatively small variations in starch composition and structure [75,76]. Moreover, the CPV ranged from 1.1 to 4.0 Pa·s. As the final viscosity, CPV reflected the stability of the swollen granule structure and was negatively correlated with the degree of long-range ordering (Figure S1). Therefore, for LRS, the ordering of the granule structure resulted in lower CPV. The resistance of the starch paste to heat could be measured by BDV, and a lower BDV value indicates better heat resistance [77]. Therefore, LRS-4 and LRS-3 exhibited better and worse resistance to heat, respectively, which could result from the diversity of ash content (Table 1 and Figure S1). The SBV ranged from 0.6 (LRS-1) to 2.6 (LRS-5) Pa·s, which could be indicative of the variety in retrogradation trend and cooking quality [64,78]. In addition, the SBV was positively correlated with AAC but negatively correlated with RC. Therefore, the SBV of LRS was mainly attributed to AAC and long-range ordered structure. It was suggested that the pasting properties of LRS resulted from a combination of multiple factors, including approximate composition, AAC, and ordered microstructure. In particular, LRS-5 showed a low gelatinization temperature, high starch paste viscosity, excellent structural stability of expanded granules, and better cooking quality. Furthermore, the differences in pasting properties among LRS samples were useful in selecting the appropriate LRS feedstocks for specific industrial applications.

### 3.8. Visual Appearance, Color, and Microstructure of Gelatinized LRS

**Figure 6:**
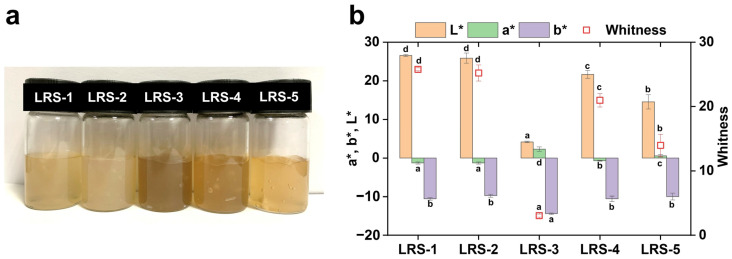
Visual observations (**a**) and color (**b**) of five gelatinized LRS samples. L*, lightness; a*, redness; b*, yellowness. Different letters in the same column indicate significant differences (*p* < 0.05).

**Figure 7:**
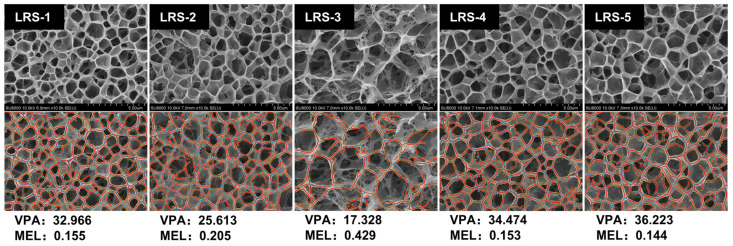
Cryo-scanning electron micrographs of five gelatinized LRS samples. The magnification was ×10.0 k. The vessels percentage area (VPA) and mean E lacunarity (MEL) data obtained by quantitative analysis with AngioTool were interpreted as the degree of network wall thickness and the looseness of the network structure, respectively.

After gelatinization, the swelling starch granules ruptured into fragments and aggregated to form a starch paste [53]. The visual appearance and color parameters of the LRS gel state are shown in Figure 6 and Table S1. The L* and whiteness values of LRS-3 showed significant differences from other samples. In addition, the a* of the LRS-3 gel sample showed the highest value, possibly related to its higher iron content (Table 2). Up to this point, the results clearly indicated that the metal components, especially iron, more greatly contribute to the redness of the gelatinized LRS samples. In general, LRS-5 exhibited an attractive gel color with moderate brightness and whiteness. The LRS-5 exhibited L* and whiteness values of 14.5 and 14.0, respectively, and the LRS-3 showed values of 4.2 and 3.1, respectively. Therefore, the LRS-5 gel had a clearer texture and lighter color than the LRS-3 gel, which might be related to the differences in iron content, and their potential application could be considered for iron-fortified foods. The microstructures of the five gelatinized LRS samples observed by cryo-SEM are presented in Figure 7. All samples exhibited a relatively uniform and continuous network structure at 10.0 k magnification. LRS-3 was the most unique, and its gel network appeared sparser and more tattered. To further compare the structural differences among the five LRS gel samples, a quantitative network analysis was performed using AngioTool software v0.5. The vessels percentage area (VPA) could be used to describe the wall thickness of the network, and the looseness of the network structure could be indicated by the mean E lacunarity (MEL). The MEL of the LRS-3 gel network structure was 0.429, and its looseness was the highest, which might be related to the highest ash content of the LRS-3 sample (Table 1). The results obtained by quantitative analysis and correlation analysis (Figure S1) showed that there was a negative correlation between the firmness of the network structure and the ash content in gelatinized LRS samples. When the ash content of starch is lower, its hydrogel structure is better at a certain concentration. In addition, Ulbrich et al. suggested that stronger gels are obtained under specific preparation conditions when the amylose content of starch is higher [79]. In the current work, the AAC of LRS was also positively correlated with the VPA of its network structure (Pearson correlation coefficient 0.6133).

### 3.9. Light Transmittance of Gelatinized LRS

**Figure 8:**
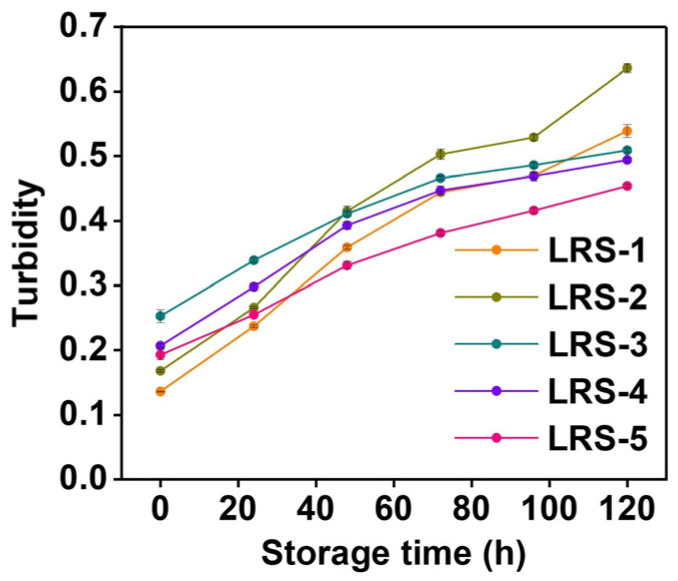
Turbidity during storage of five gelatinized LRS samples.

Typically, the light transmittance of gelatinized starch is mainly related to the degree by which starch undergoes molecular rearrangement after gelatinization and retrogradation, which could reflect the retrograde trend of starch hydrogels during short-term storage [62]. A higher rearrangement degree generally led to lower light transmittance, indicating a higher degree of retrogradation. Turbidity was used as a light transmittance evaluation index of gelatinized LRS. When the turbidity of the gelatinized LRS was lower, the light transmittance of the sample was higher, which leads to a better appearance and luster of the system and a better expected appearance of the developed products. The development of paste turbidity in the LRS samples is shown in Figure 8. The lowest turbidity appeared at 0 h for all samples. Notably, LRS-5 gradually presented the advantage of light transmittance during 120 h of low-temperature storage. The retrogradation trend observed for LRS-2 was the most obvious, especially after 48 h. In general, the main factors affecting the light transmittance of starch-based materials during storage include solubility, AAC, short- and long-range ordered structure, granule swelling, and intramolecular and intermolecular interactions [30]. According to correlation analysis (Figure S1), the turbidity of LRS samples that had recently undergone gelatinization was mainly positively correlated with ash content (Pearson correlation coefficient 0.77581) but negatively correlated with solubility (−0.78678). However, the turbidity of gelatinized LRS after 5 days of storage was more related to the AAC and structural characteristics of starch granules. Therefore, the ordered structure might be the main factor to promote the retrogradation of LRS during short-term storage.

### 3.10. Freeze-Thaw Stability of Gelatinized LRS

**Figure 9:**
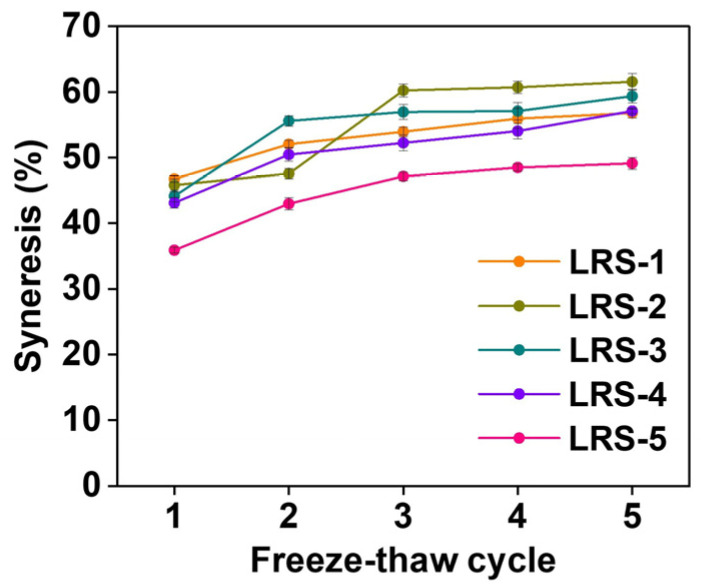
Syneresis in 5 freeze-thaw cycles of 5 gelatinized LRS samples.

Freeze-thaw stability is among the critical factors that reflects the stability of the food quality of starch-based foods during temperature changes [80]. Syneresis could reflect the steadiness of the system structure by the amount of water released from the gel. As shown in Figure 9, the stability of five gelatinized LRS samples in five freeze-thaw cycles was compared by syneresis. The smaller the syneresis was, the better the freeze-thaw stability. With the increase in the cycle number, the syneresis of the LRS gradually increased. The increase in syneresis resulted from random interactions that reduced the water retention of starch gels after freezing and thawing [81]. With the swelling of the starch granules, the intermolecular and intramolecular hydrogen bonds increase due to interchain interactions, which increases the distance between molecular chains and facilitates water discharge during the thawing process [30]. The freeze-thaw stability of LRS-5 was relatively stable and optimal, and compared to other samples, it showed more significant potential as a base material for frozen food. The resulted indicated that the microstructure of LRS-5 gel exhibited a denser arrangement, which contributed significantly to freeze-thaw stability. Overall, the freeze-thaw stability and short-term storage turbidity trends were significantly correlated with the AAC and structural characteristics of LRS (Figure S1). Specifically, more AAC and a lower degree of short- and long-range ordered structures resulted in better freeze-thaw stability and light transmittance, which might point to lower short-term retrogradation levels [82].

## 4. Conclusions

LRS from different regions exhibited varying physicochemical properties, mainly depending upon varied approximate compositions and structural characteristics. The ash content, AAC, and ordered structure were significantly correlated with the physicochemical properties of granular and gelatinized LRS. LRS with more intact and smooth granule morphology and higher AAC exhibited better swelling, gelatinization viscosity, network structure firmness, light transmittance, and freeze-thaw stability. In comparison, LRS with higher ash content, especially iron content, showed less RS, lower swelling power and gelatinization temperature, a darker gel color, and looser microstructure. The differences in thermal and pasting properties among LRS samples were probably the result of a combination of multiple factors, including starch granule morphology, ash content, AAC, as well as short- and long-range ordered structure. In addition, LRS was resistant to *in vitro* enzymatic digestion, with a digestibility of less than 23%, among which LRS-5 contained the most RS, mainly due to its smoother granule surface, lower fragmentation, and higher AAC. In conclusion, this study could provide a theoretical basis for the appropriate applications of LRS as a hydrogel matrix in future food realms, such as nutrition enhancement and additive manufacturing, through the provision of knowledge on the physicochemical properties of LRS from different regions.

## Figures and Tables

**Table 1:** Proximate composition and apparent amylose content (AAC) of five LRS samples.

Sample	Moisture Content (g/kg)	Starch Content (g/kg)	Protein (g/kg)	Fat (g/kg)	Ash (g/kg)	AAC (g/kg)
LRS-1	82.18 ± 0.27 ^b^	796.75 ± 63.25 ^ab^	1.61 ± 0.14 ^a^	0.62 ± 0.26 ^a^	4.00 ± 0.00 ^a^	283.74 ± 5.41 ^b^
LRS-2	89.07 ± 1.04 ^c^	718.77 ± 9.50 ^a^	1.83 ± 0.00 ^ab^	1.65 ± 1.18 ^a^	3.83 ± 0.29 ^a^	265.79 ± 1.83 ^a^
LRS-3	74.36 ± 0.55 ^a^	739.73 ± 13.54 ^a^	1.88 ± 0.26 ^ab^	4.35 ± 2.40 ^a^	11.83 ± 0.29 ^c^	282.51 ± 4.39 ^b^
LRS-4	91.01 ± 2.58 ^c^	853.27 ± 45.06 ^b^	2.17 ± 0.08 ^b^	3.62 ± 1.85 ^a^	3.50 ± 0.00 ^a^	294.91 ± 4.10 ^c^
LRS-5	120.30 ± 0.62 ^d^	886.68 ± 45.82 ^b^	1.86 ± 0.07 ^ab^	1.08 ± 0.16 ^a^	5.00 ± 0.00 ^b^	316.49 ± 2.28 ^d^

Data are reported as means ± SD from triplicate determinations. Different letters in the same column indicate significant differences (*p* < 0.05). AAC, apparent amylose content.

**Table 2:** Main metal contents of five LRS samples.

Sample	Calcium (mg/kg)	Iron (mg/kg)	Magnesium (mg/kg)	Manganese (mg/kg)
LRS-1	486.34 ± 9.72 ^c^	84.57 ± 1.99 ^c^	33.94 ± 0.72 ^a^	2.24 ± 0.04 ^a^
LRS-2	411.51 ± 13.10 ^b^	53.68 ± 0.58 ^b^	44.95 ± 0.34 ^c^	2.31 ± 0.14 ^a^
LRS-3	354.49 ± 5.31 ^a^	302.60 ± 3.63 ^e^	127.05 ± 2.61 ^e^	3.91 ± 0.03 ^b^
LRS-4	479.30 ± 9.16 ^c^	50.57 ± 1.37 ^a^	37.38 ± 1.76 ^b^	2.37 ± 0.08 ^a^
LRS-5	411.61 ± 2.48 ^b^	125.57 ± 2.14 ^d^	50.50 ± 0.95 ^d^	7.24 ± 0.04 ^c^

Data are reported as means ± SD from triplicate determinations. Different letters in the same column indicate significant differences (*p* < 0.05).

**Table 3:** Digestion parameters of five LRS samples.

Sample	RDS (%)	SDS (%)	RS (%)
LRS-1	26.49 ± 1.79 ^c^	26.56 ± 2.97 ^b^	46.95 ± 2.69 ^c^
LRS-2	30.47 ± 4.64 ^c^	29.51 ± 6.16 ^b^	40.03 ± 3.49 ^b^
LRS-3	40.63 ± 0.47 ^d^	40.10 ± 2.79 ^c^	15.27 ± 2.32 ^a^
LRS-4	24.18 ± 0.33 ^b^	23.31 ± 0.09 ^b^	52.50 ± 0.43 ^d^
LRS-5	12.44 ± 0.91 ^a^	17.92 ± 1.58 ^a^	69.65 ± 2.18 ^e^

Data are reported as means ± SD from triplicate determinations. Different letters in the same column indicate significant differences (*p* < 0.05). RDS, rapidly digested starch; SDS, slowly digested starch; RS, resistant starch.

**Table 4:** Structural characteristic parameters of five LRS samples.

Sample	R_1045/1022_	R_1022/995_	RC (%)
LRS-1	0.65 ± 0.03 ^b^	1.53 ± 0.02 ^d^	27.34 ± 0.62 ^c^
LRS-2	0.73 ± 0.01 ^c^	1.22 ± 0.01 ^a^	29.37 ± 0.48 ^d^
LRS-3	0.66 ± 0.02 ^b^	1.45 ± 0.00 ^c^	26.57 ± 0.09 ^c^
LRS-4	0.63 ± 0.02 ^b^	1.37 ± 0.03 ^b^	24.75 ± 0.44 ^b^
LRS-5	0.58 ± 0.00 ^a^	1.59 ± 0.05 ^e^	21.40 ± 0.70 ^a^

Data are reported as means ± SD from triplicate determinations. Different letters in the same column indicate significant differences (*p* < 0.05). RC, relative crystallinity.

**Table 5:** Thermal characteristics of five LRS samples.

Sample	*T*_o_ (°C)	*T*_p_ (°C)	*T*_c_ (°C)	Δ*H*_gel_ (J/g)
LRS-1	56.48 ± 0.09 ^b^	68.93 ± 0.31 ^d^	80.34 ± 0.16 ^a^	5.50 ± 0.06 ^bc^
LRS-2	56.29 ± 0.13 ^b^	67.48 ± 0.22 ^c^	88.23 ± 0.61 ^d^	4.81 ± 0.13 ^a^
LRS-3	53.26 ± 0.31 ^a^	63.72 ± 0.40 ^a^	79.49 ± 0.55 ^a^	5.31 ± 0.15 ^b^
LRS-4	59.98 ± 0.15 ^d^	68.72 ± 0.48 ^d^	83.55 ± 0.96 ^c^	5.30 ± 0.21 ^bc^
LRS-5	56.90 ± 0.17 ^c^	65.00 ± 0.10 ^b^	81.32 ± 0.20 ^b^	6.09 ± 0.46 ^c^

Data are reported as means ± SD from triplicate determinations. Different letters in the same column indicate significant differences (*p* < 0.05). *T*_o_, *T*_p,_ and *T*_c_ represent the onset, peak, and conclusion temperatures, respectively, and Δ*H*_gel_ is the enthalpy for starch melting.

**Table 6:** Pasting properties of five LRS samples.

Sample	PT (°C)	PV (Pa·s)	HPV (Pa·s)	CPV (Pa·s)	BDV (Pa·s)	SBV (Pa·s)
LRS-1	67.71 ± 1.93 ^bc^	1.32 ± 0.04 ^a^	0.43 ± 0.01 ^a^	1.06 ± 0.07 ^a^	0.89 ± 0.03 ^b^	0.63 ± 0.06 ^a^
LRS-2	66.05 ± 0.97 ^b^	1.67 ± 0.15 ^b^	0.71 ± 0.04 ^b^	1.80 ± 0.15 ^b^	0.97 ± 0.15 ^bc^	1.10 ± 0.14 ^b^
LRS-3	62.58 ± 0.97 ^a^	2.30 ± 0.11 ^c^	1.15 ± 0.08 ^c^	3.07 ± 0.13 ^c^	1.15 ± 0.15 ^c^	1.92 ± 0.05 ^c^
LRS-4	69.90 ± 0.04 ^c^	1.65 ± 0.12 ^b^	1.05 ± 0.04 ^c^	3.17 ± 0.30 ^cd^	0.60 ± 0.08 ^a^	2.12 ± 0.25 ^c^
LRS-5	63.82 ± 0.95 ^a^	2.26 ± 0.18 ^c^	1.33 ± 0.07 ^d^	3.97 ± 0.43 ^d^	0.93 ± 0.11 ^bc^	2.64 ± 0.37 ^d^

Data are reported as means ± SD from triplicate determinations. Different letters in the same column indicate significant differences (*p* < 0.05). PT, pasting temperature; PV, peak viscosity; HPV, hot paste viscosity; CPV, cold paste viscosity; BDV, breakdown viscosity (BDV = PV − HPV); SBV, setback viscosity (SBV = CPV − HPV).

## Data Availability

The data presented in this study are available on request from the corresponding author.

## Supplementary Materials

The following supporting information can be downloaded at: https://www.mdpi.com/article/10.3390/foods12234330/s1, Table S1: Color of five LRS samples; Figure S1: Principal component analysis (a, 95% confidence ellipse) and Pearson correlation coefficients (b, * *p* < 0.05) of the basic physicochemical of LRS; Figure S2: Thermal properties (a) and pasting properties (b and c) curves of five LRS samples.
