# Supplementary material for: Physicochemical Properties of Granular and Gelatinized Lotus Rhizome Starch with Varied Proximate Compositions and Structural Characteristics
Source: Foods. 2023 Nov 30;12(23):4330. doi: 10.3390/foods12234330 (PMC10706325; doi:10.3390/foods12234330)
Supplement: Supplementary file 1 [file foods-12-04330-s001.zip › foods-2714500-supplementary.pdf]

Supplementary

Physicochemical properties of granular and gelatinized lotus rhizome starch with varied proximate compositions and structural characteristics

Xin-Yu Jiang [1], Yi-Ting Gu [1], Lichao Zhang [1, 2], Jin-Jian Sun [3], Jia-Nan Yan [1], Ce Wang [1], Bin Lai [1] and Hai-Tao Wu [1*]

[1] SKL of Marine Food Processing & Safety Control, National Engineering Research Center of Seafood, Collaborative Innovation Center of Seafood Deep Processing, School of Food Science and Technology, Dalian Polytechnic University, Dalian 116034, China;

[2] Institutes of Biomedical Sciences, Shanxi University, Taiyuan 030006, China;

[3] Dalian Center for Food and Drug Control and Certification, Dalian 116037, China.

* Correspondence: wht205@163.com (H. W.); Tel: 86-411-86318731; Fax: 86-411-86318655

Table S1. Color of five LRS powder and gel

Sample	Powder				Gel			
	**L***	**a***	**b***	**Whiteness**	**L***	**a***	**b***	**Whiteness**
LRP-1	89.58±0.73[b]	3.07±0.04[b]	9.66±0.20[a]	85.46±0.67[c]	26.57±0.25[d]	-1.28±0.28[a]	-10.57±0.33[b]	25.80±0.23[d]
LRP-2	89.29±0.57[b]	3.44±0.10[cd]	10.70±0.19[c]	84.47±0.32[bc]	25.88±1.30[d]	-1.27±0.28[a]	-9.78±0.38[b]	25.22±1.26[d]
LRP-3	83.45±0.57[a]	3.48±0.04[c]	10.18±0.10[b]	80.26±0.45[a]	4.15±0.22[a]	2.28±0.56[d]	-14.43±0.19[a]	3.05±0.24[a]
LRP-4	88.48±0.32[b]	3.62±0.06[d]	10.12±0.12[b]	84.24±0.24[b]	21.66±0.99[c]	-0.69±0.12[b]	-10.57±0.70[b]	20.95±1.05[c]
LRP-5	89.19±0.54[b]	2.92±0.05[a]	10.75±0.12[c]	84.47±0.30[bc]	14.54±1.83[b]	0.57±0.32[c]	-9.99±0.88[b]	13.95±1.73[b]

Data are reported as means±SD from triplicate determinations. Different letters in the same column indicate significant differences (*P*<0.05). L*, lightness; a*, redness; b*, yellowness.

a

b

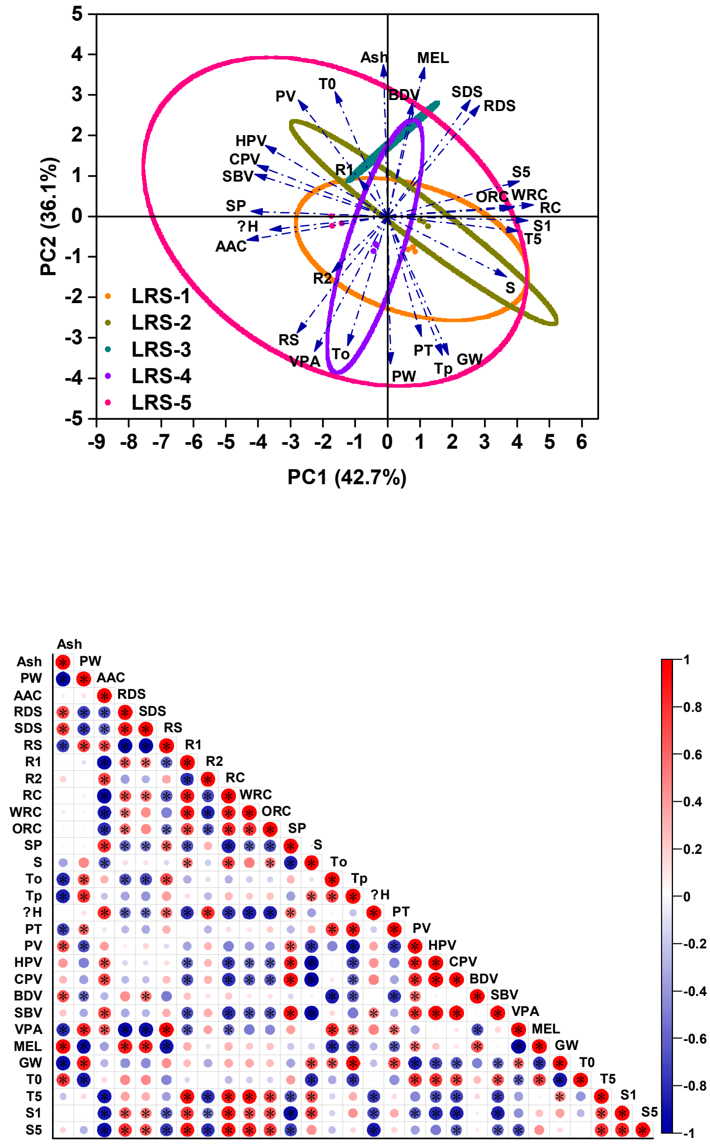

Figure S1. Principal component analysis (a, 95% confidence ellipse) and Pearson correlation coefficients (b, *P<0.05) and between the basic physicochemical and functional properties of LRS. PW, powder whiteness; AAC, apparent amylose content; RDS, rapidly digested starch; SDS, slowly digested starch; RS, resistant starch; R1, $R_{1045/1022}$; R2, $R_{1022/995}$; RC, relative crystallinity; WRC, water retention capacity; ORC, oil retention capacity; SP, swelling power at 90 °C; S, solubility at 90 °C; To, onset gelatinization temperature; Tp, peak gelatinization temperature; ΔH, enthalpy for starch gelatinization; PT, pasting temperature; PV, peak viscosity; HPV, hot paste viscosity; CPV, cold paste viscosity; BDV, breakdown viscosity; SBV, setback viscosity; VPA, vessels percentage area; MEL, mean E lacunarity; GW, gel whiteness; T0, turbidity at day 0; T5, turbidity at day 5; S1, syneresis of the first freeze-thaw cycle; S5, syneresis of the 5th freeze-thaw cycle.

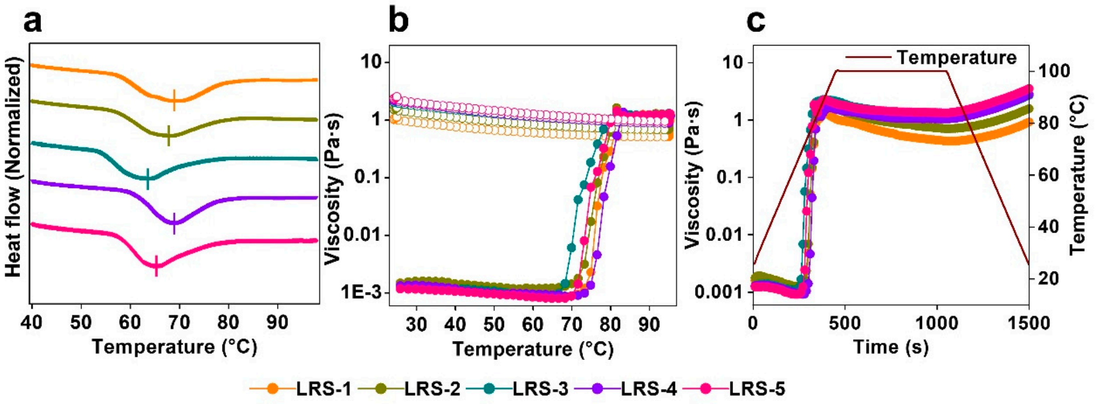

Figure S2. Thermal properties (a) and pasting properties (b and c) curves of five LRS samples.
